# Remimazolam sedation with regional anesthesia in difficult airway due to huge thyroid goiter: A case report

**DOI:** 10.1097/MD.0000000000035497

**Published:** 2023-10-20

**Authors:** Gwanbeom Kim, Yu Yil Kim, Hyun Joo Heo, Junyoung Park

**Affiliations:** a Department of Anesthesiology and Pain Medicine, Presbyterian Medical Center, Jeonju, Jeollabuk-Do, Korea.

**Keywords:** anesthesia, case report, difficult airway, goiter, remimazolam, sedation

## Abstract

**Rationale:**

Remimazolam, a novel benzodiazepine, is known to have less respiratory depression compared to other anesthetic agents, and it also has a reversal agent that can be used in emergency situations. Remimazolam with these characteristics can be usefully utilized in the anesthetic management of patients with difficult airway.

**Patient concerns:**

A 78-year-old female patient was scheduled proximal humerus fracture surgery. The patient occasionally complained dyspnea and had multiple comorbidities including thyroid goiter, dementia, and delirium.

**Diagnoses:**

The patient had a large thyroid goiter compressing and deviating the trachea. A short neck with increased circumference was confirmed. A difficult airway was anticipated in the preanesthetic evaluation.

**Interventions:**

Sedation with remimazolam followed by regional anesthesia was performed for the surgery.

**Outcomes:**

The surgery was completed without complications. The patient recovered and was discharged on 15th postoperative days.

**Lessons:**

The use of remimazolam for sedation may be an appropriate option in the anesthetic management of patients with difficult airway.

## 1. Introduction

Airway management is a critical aspect of anesthesia. On the condition that preanesthetic evaluation suggests difficulty with airway management, it is essential to establish a safe and appropriate anesthesia plan in advance. Regional anesthesia, sedation, or general anesthesia may be considered depending on the patient’s condition and the type of surgery. When under general anesthesia, difficult airway is predicted, it is important to consider options for airway management, such as awake intubation or surgical airway access. In cases where sedation is needed under regional anesthesia, proper management for self-breathing should be ensured.

Commonly used intravenous anesthetics include midazolam, propofol, dexmedetomidine, and remimazolam. Among these, dexmedetomidine and remimazolam are known to have lower respiratory depression rates than propofol,^[1,2]^ making them suitable options for difficult airway management. Remimazolam is a new benzodiazepine that has hemodynamic safety and lower respiratory depression risk compared to other intravenous anesthetics.^[1,3,4]^ Additionally, it can be easily reversed by flumazenil.^[5]^ Therefore, it can be useful option for difficult airway management. In this report, we present a case of a 78-year-old female patient with a large thyroid goiter compressing the trachea, who underwent successful proximal fracture surgery under sedation with remimazolam and regional anesthesia. Along with this literature review, we provide insights into the safe and effective use of remimazolam.

## 2. Case presentation

### 2.1. Patient information

**Figure 1.**
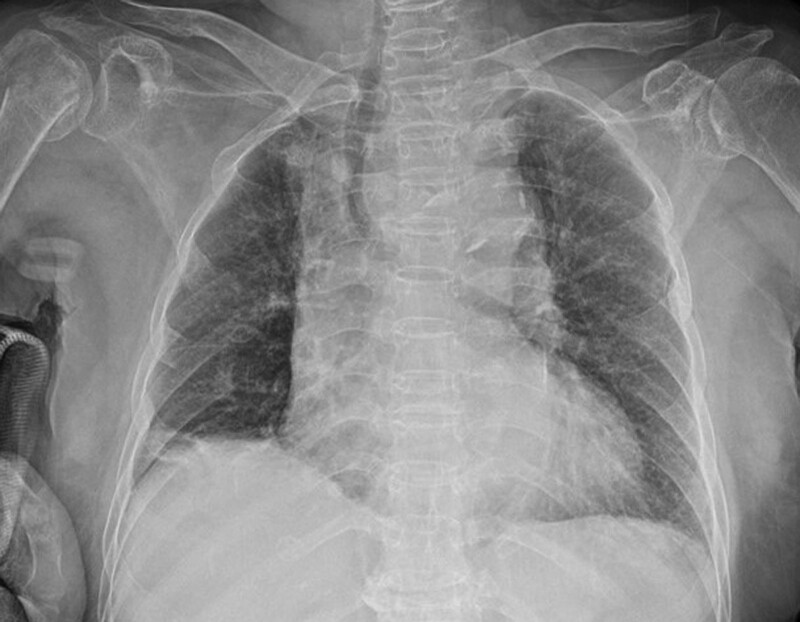
Chest radiography. Trachea is deviated to the right.

**Figure 2.**
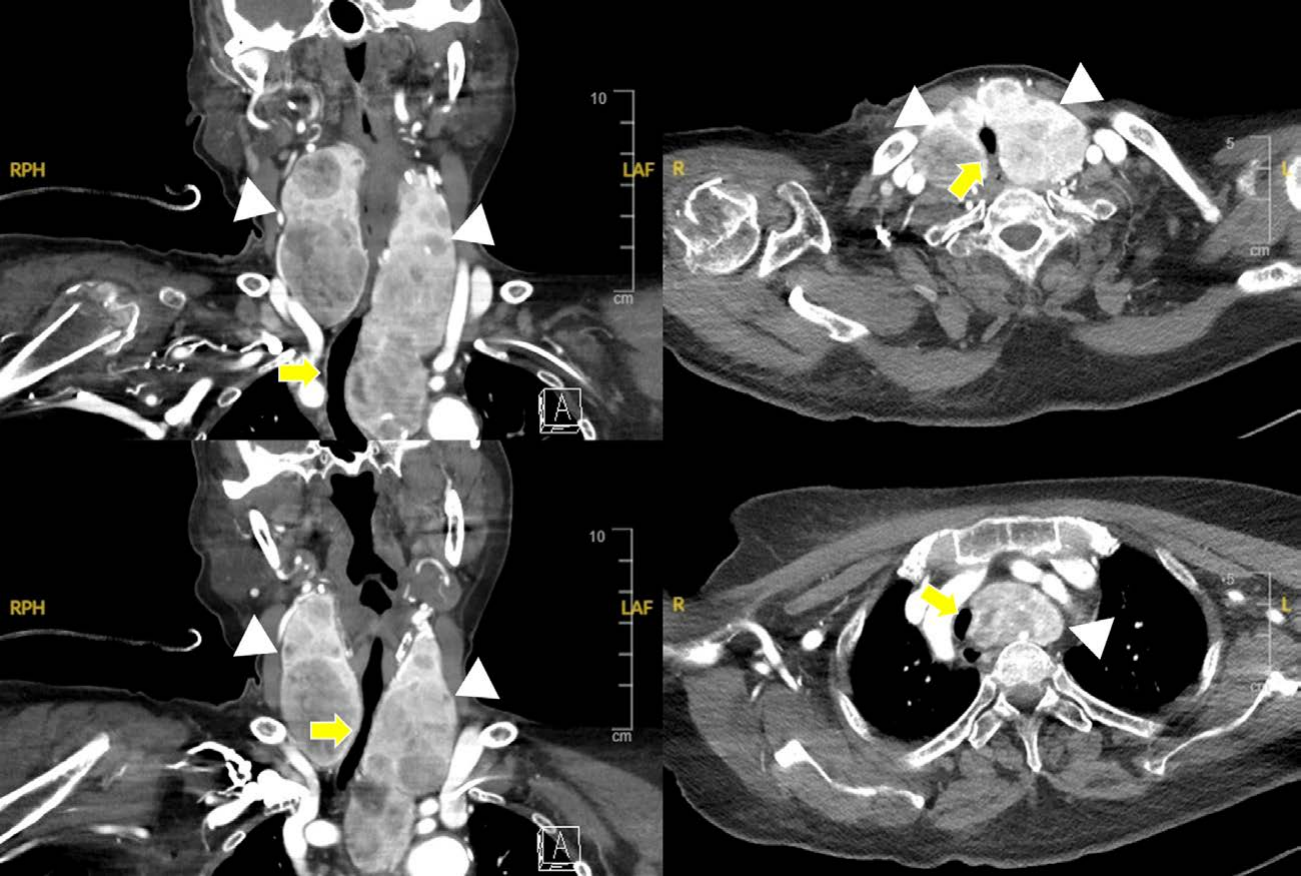
Neck computed tomography. A huge multinodular goiter (white arrow head) extends into the retrosternal region and compresses the trachea (yellow arrow), causing it to be deviated to the right.

A 78-years old female (145 cm/60 kg, body mass index 28.5) was admitted to our hospital for proximal humerus fracture and scheduled for open reduction and internal fixation. The patient had a medical history including huge thyroid goiter, dementia, depression, diabetes mellitus, asthma and osteoporosis. The patient was diagnosed with thyroid goiter and hyperthyroidism 6 years ago and was treated with methimazole. Thyroid function tests have been within the normal range for the past 2 years. Trachea was deviated to the right side in chest radiography (Fig. 1). The preanesthetic neck computed tomography revealed that the right thyroid lobe was measured 9.0 × 4.3 × 4.4 cm and the left thyroid lobe was measured 13.9 × 4.1 × 4.7 cm. Trachea was compressed and deviated to the right side (Fig. 2). The shortest diameter of trachea was 0.66 cm at a point 7.5 cm below vocal cord. The patient occasionally complained of mild dyspnea, and 2 L/minutes of oxygen was supplied through nasal prong after admission. Thyroid function tests, electrocardiogram and arterial blood gas analysis are within normal range in preanesthetic evaluation. Delirium with violent behavior occurred in the patient before surgery. The patient suffered from aggressive delirium for several weeks while hospitalized for femur fracture surgery 2 years ago. Proper airway assessment including mallampati classification, thyromental distance, neck extension, mouth opening could not be conducted due to delirium in the preanesthetic evaluation. However, a short neck with increased circumference (41.2 cm) was confirmed.

### 2.2. Anesthetic planning

Airway management during anesthesia was predicted to be difficult in the preanesthetic evaluation. Therefore, we established an anesthesia management plan as follows to minimize airway-associated hazards; Regional anesthesia, interscalene brachial plexus block, is performed as a first-line strategy. Sedation is performed prior to regional anesthesia while the patient is unable to cooperate during regional anesthesia and surgery due to dementia and delirium. Remimazolam is used as a sedative agent, which has less respiratory depression and can be reversed in emergencies. Supraglottic airway device, video-laryngoscope, and fiberoptic bronchoscope are prepared as an emergency airway management devices.

### 2.3. Anesthetic management

Upon arrival in the operating room, standard monitoring devices including noninvasive blood pressure, electrocardiogram, pulse oximetry, body temperature, patient state index (PSI) were attached to the patient. Initial noninvasive blood pressure was 160/70 mm Hg, Heart rates was 90 beats/minutes, and oxygen saturation was 93% in room air. Oxygen was administered via a facial mask at a rate of 5 L/minutes. Remimazolam (Byfavo, Hana pharm Co., Hwaseong, Korea) was started at 1.5 mg/kg/hour. After the loss of consciousness, remimazolam was adjusted at a rate of 0.4 to 1.0 mg/kg/hour to maintain PSI 50 to 70. Respiration of the patient was monitored by electrocardiogram and end-tidal CO_2_. Regional anesthesia was performed by brachial plexus block via interscalene approach. Ultrasound and nerve stimulator were used for nerve block. In left semi-lateral position, aseptic maneuver was done. The cervical structures were deviated posteriorly by the enlarged thyroid mass on the ultrasound. After confirming the trunk of the brachial plexus with ultrasound lying postero-lateral to the subclavian artery at the supraclavicular fossa, a probe was moved along the nerve to the cephalad, and 1:1 solution of 2% lidocaine and 0.75% ropivacaine was administered at interscalene region. A total of 28 mL of local anesthetic was injected. The absence of response to painful stimuli was confirmed, and then surgery proceeded in the sitting position.

The operation time and the sedation time were 93 minutes and 105 minutes, respectively. The dose of remimazolam for loss of consciousness was 8 mg, and the total dose used for sedation was 75 mg. PSI was maintained between 44 to 66 during regional anesthesia and surgery. Oxygen saturation maintained at 94% to 98%. The respiratory rate was 15 to 25 times/minutes and apnea was not observed. No airway manipulation were performed. Remimazolam administration was discontinued when the subcutaneous tissue was being closed. After the completion of the surgery, flumazenil 0.3 mg was administered for reverse the effects of remimazolam. The patient was transferred to the intensive care unit after surgery and was transferred to a general ward the next day. Delirium improved 7th postoperative days, and patient was discharged on 15th postoperative days.

## 3. Discussion

We report a successful surgery for humerus fracture using regional anesthesia and sedation with remimazolam in a patient with a huge thyroid mass that was predicted difficult airway.

There is no perfect method to predict airway management difficulty. Facial features assessments (mouth opening, head and neck mobility, presence of a beard, etc), anatomical measurements and landmarks assessments (Mallampati scores, thyromental distance, neck circumference, ratio of neck circumference to thyromental distance, etc), and additional evaluations (airway ultrasound, bedside endoscopy, 3-dimensional printing, etc) have been used as reliable methods for predicting difficult airways.^[6]^ There is ongoing debate regarding the relationship between large thyroid goiters and difficult airways.^[7–10]^ Although thyroid goiter is not a factor that directly cause difficulty in airway management, it can lead to increased challenges in airway management due to increased neck circumference, limited mouth opening, and restricted neck extension.^[8,10,11]^ Moreover, huge thyroid goiters can result in anatomical changes that make invasive airway access, such as emergency tracheostomy or cricothyrotomy, impossible. Therefore, considering patient factors and surgical considerations, an appropriate anesthesia and corresponding airway management plan should be established prior to anesthesia in patients with huge thyroid goiter.

Both general anesthesia and regional anesthesia can be used for proximal humerus surgery. When anticipating a difficult airway, options such as awake intubation or invasive access may need to be considered for general anesthesia.^[12]^ On the other hand, regional anesthesia can also be a useful option,^[13]^ and it is known to have a better outcome including safety, postoperative pain control, and patient satisfaction in proximal humerus surgery.^[12,14]^ In this case, we decided that regional anesthesia would be the proper anesthetic method. Patient cooperation is crucial for regional anesthesia. However, dementia and delirium are factors that interfere with regional anesthesia and surgery. Therefore, we decided to perform sedation before regional anesthesia. It is important to note that sedation can produce detrimental effects. In particular, oversedation is associated with a risk of respiratory depression, airway obstruction, hypoxia, and aspiration. Therefore, an independent anesthesiologist delivering, monitoring, and titrating sedation is recommended, and Dexmedetomidine and remifentanil are associated with low risk of oversedation.^[15]^ Additionally, remimazolam can also be used as a sedative drug.

Remimazolam is a novel benzodiazepine used for general anesthesia and procedural sedation. It combines the properties of 2 existing drugs used in anesthesia: midazolam and remifentanil. Remimazolam modulates the effects of γ-aminobutyric acid (GABA) type A receptor in order to enhance the effects of GABA. Remimazolam is a rapidly metabolized by nonspecific esterases into an active metabolite that has no clinically relevant effects at the receptor site. Therefore, remimazolam exhibits rapid on-off set. Remimazolam has less hemodynamic and respiratory depression than propofol.^[1]^ Recent study comparing remimazolam and dexmedetomidine have shown similar effects of respiratory depression.^[16]^ Even in cases where remimazolam occurred higher respiratory depression than dexmedetomidine, manual ventilation was not required.^[17]^ Additionally, a significant advantage of remimazolam is the availability of a specific reversal agent flumazenil, which can be used in cases of oversedation or emergency situations. Flumazenil works by binding to the same receptors in the brain that benzodiazepines bind to, but it has the opposite effect. Rather than enhancing the effects of GABA, flumazenil blocks the activity of benzodiazepines and can quickly reverse their sedative effects.^[5]^ Therefore, in this case, remimazolam was used sedative drug, and the operation was successfully completed without events and complications.

## 4. Conclusion

Remimazolam provided adequate sedation without respiratory-related adverse events in patient with anticipated difficult airway. It can be considered as a sedative agent for patients with difficult airways, and further prospective studies should be conducted to confirm its use in this context.

## Author contributions

**Conceptualization:** Yu Yil Kim.

**Data curation:** Hyun Joo Heo.

**Investigation:** Gwanbeom Kim, Junyoung Park.

**Supervision:** Yu Yil Kim.

**Writing – original draft:** Gwanbeom Kim, Yu Yil Kim.

**Writing – review & editing:** Yu Yil Kim, Hyun Joo Heo.
